# Salidroside Derivative SHPL-49 Exerts Anti-Neuroinflammatory Effects by Modulating Excessive Autophagy in Microglia

**DOI:** 10.3390/cells14060425

**Published:** 2025-03-13

**Authors:** Zhirui Zheng, Ruyi Wang, Yu Zhao, Pei Zhang, Dong Xie, Shoujiao Peng, Ruixiang Li, Jiange Zhang

**Affiliations:** The Research Center of Chiral Drugs, Innovation Research Institute of Traditional, Chinese Medicine (IRI), Shanghai University of Traditional Chinese Medicine, No. 1200 Cailun Road, Zhangjiang Hi-Tech Park, Shanghai 201203, China

**Keywords:** ischemic stroke, autophagy, neuroinflammation, microglia, rhodioloside derivative

## Abstract

The neuroinflammation triggered by cellular demise plays a pivotal role in ameliorating the injury associated with ischemic stroke, which represents a significant global burden of mortality and disability. The compound SHPL-49, a derivative of rhodioloside, was discovered by our research team and has previously demonstrated neuroprotective effects in rats with ischemic stroke. This study aimed to elucidate the underlying mechanisms of SHPL-49’s protective effects. Preliminary investigations revealed that SHPL-49 effectively alleviates PMCAO-induced neuroinflammation. Further studies indicated that SHPL-49 downregulates the expression of the lysosomal protein LAMP-2 and reduces lysosomal activity, impeding the fusion of lysosomes and autophagosomes, thus inhibiting excessive autophagy and increasing the expression levels of the autophagy proteins LC3-II and P62. Furthermore, SHPL-49 effectively reverses the NF-κB nuclear translocation induced by the autophagy inducer rapamycin, significantly lowering the expression levels of the inflammatory factors IL-6, IL-1β, and iNOS. In a co-culture system of BV2 and PC12 cells, SHPL-49 enhanced PC12 cell viability by inhibiting excessive autophagy in BV2 cells and reducing the ratio of apoptotic proteins Bax and BCL-2. The overall findings suggest that SHPL-49 exerts its neuroprotective effects through the inhibition of excessive autophagy and the suppression of the NF-κB signaling pathway in microglia, thereby attenuating neuroinflammation.

## 1. Introduction

Stroke, the second leading cause of mortality worldwide, is responsible for approximately 5.5 million deaths annually and continues to be a significant contributor to global disability [1]. Stroke is primarily classified into hemorrhagic and ischemic subtypes, with ischemic strokes accounting for up to 71% [2]. Following the impact of ischemia, a cascade of interconnected cytoplasmic and nuclear events occurs at the damaged site, leading to bioenergetic depletion, excitotoxicity, excessive calcium influx, oxidative stress, and inflammatory responses that ultimately result in neuronal cell death [3]. The inflammation caused by cell death can further aggravate the injury by impairing the ischemic penumbra through bystander effects. The mounting evidence suggests that post-ischemic inflammation plays a pivotal role in the progression of brain injury. The severity of outcomes in acute ischemic stroke is significantly influenced by the magnitude of this inflammatory response [4,5]. The appropriate termination of neuroinflammation after a stroke has been demonstrated to effectively promote tissue repair and regeneration, thereby offering considerable therapeutic benefits [6].

The microglia, as the resident immune cells of the central nervous system [7], are indispensable inflammatory cells that contribute to maintaining homeostasis and play an important role in neuroinflammation and neurodegenerative diseases [8]. Following the onset of ischemic stroke, microglia can promote tissue repair through the clearance of damaged cells or debris and the production of anti-inflammatory cytokines and growth factors [9,10]. The cells can also release proinflammatory cytokines, such as IL-6, IL-1β, and iNOS, concurrently contributing to the inflammatory response [11,12,13]. The central role of microglia in neuroinflammation renders them a viable therapeutic target for modulating post-ischemic stroke inflammation [14]. The NF-κB signaling pathway is a canonical cytokine-mediated signaling cascade that plays a crucial role in inflammatory processes [15]. Consequently, the inhibition of NF-κB activation may represent a potential strategy for mitigating inflammation and safeguarding against brain damage.

The process of autophagy plays a pivotal role in the innate immune response and functions as an adaptive mechanism for cells to adapt to metabolic stress and environmental fluctuations [16,17]. It facilitates the degradation or elimination of damaged proteins and organelles within the cytoplasm through the lysosomal degradation pathway, thereby ensuring cellular homeostasis. When triggered by diverse stress conditions, autophagy safeguards cell survival and homeostasis by clearing protein aggregates and dysfunctional mitochondria, recovering amino acids, fatty acids, and glucose to sustain energy balance while mitigating inflammation [18]. The mild-to-moderate activation of autophagy can, thus, serve as a cellular pro-survival mechanism. However, excessive or prolonged autophagic activation may ultimately result in cell death [19]. The excessive accumulation of autophagosomes may necessitate the exacerbation of cellular component degradation, leading to cellular injury and the increased secretion of inflammatory factors [20,21,22]. Therefore, exploring autophagy in the context of ischemic stroke challenges may provide innovative strategies for stroke prevention and treatment.

Autophagy and inflammation are two critical, interconnected processes by which microglia execute their functions during ischemic stroke. Under normal circumstances, autophagy is considered to exert a regulatory effect on microglial inflammation; however, under activated conditions, it may serve a dual role as both an inhibitory and stimulatory factor in ischemic inflammation [23]. The application of autophagy inhibitors, for instance, enhances the expression of inflammatory genes in adipocytes; conversely, the activation of autophagy reduces the expression of inflammatory genes, thereby indicating that autophagy has the capacity to modulate inflammatory responses [24]. The use of autophagy inhibitors, such as 3-methyladenine, conversely impedes autophagic activity and alleviates LPS-induced acute lung injury by suppressing inflammation and reversing autophagy. This suggests that the inhibition of autophagy may also mitigate inflammatory responses [25]. The effective regulation of the interplay between microglial autophagy and inflammation may offer novel avenues for research aimed at enhancing protection against ischemic stroke.

The novel compound SHPL-49, developed by our team as a structurally modified derivative of salidroside, has exhibited neuroprotective effects against ischemic stroke [26,27,28]. The pharmacological effects of SHPL-49 were initially discovered to exert neuroprotective properties through the inhibition of calcium influx, oxidative stress injury, apoptosis, and other mechanisms. This subsequently leads to a reduction in cerebral infarction volume and effectively alleviates ischemic brain injury [27]. After further investigation into the mechanism of action and potential targets of SHPL-49, we have discovered that SHPL-49 exhibits the ability to enhance neuronal survival and alleviate acute ischemic stroke by facilitating the NR2A-CAMKIIα-Akt/CREB pathway [28]. Moreover, our findings indicate that SHPL-49 can activate angiogenesis via the microglia-mediated VEGFR2/Akt/eNOS pathway and suppress the p38 MAPK/MMP-9 signaling cascade to mitigate blood–brain barrier (BBB) disruption in microglia, thereby exerting neuroprotective effects [29]. These results indicate that microglia potentially exert a crucial influence on regulating the neuroprotective effects of SHPL-49 in cases of ischemic stroke. Additionally, our study demonstrated that the regulation of microglia by SHPL-49 induces the secretion of neurotrophic factors while reducing the release of inflammatory mediators. Ultimately, SHPL-49 attenuates neuronal damage caused by ischemia and promotes neurogenesis through the activation of the BDNF/TrkB/Gap43 pathway, thereby enhancing synaptic plasticity [26]. Although our study demonstrated the potential of SHPL-49 in mitigating microglia-induced neuroinflammation, the precise underlying mechanisms remain elusive and warrant further investigation. In light of the pivotal regulatory roles played by microglia and autophagy in the neuroinflammatory response triggered by ischemic stroke, this study aims to investigate whether SHPL-49 can ameliorate the occurrence of neuroinflammation through the modulation of microglial autophagy, thereby conferring neuronal protection. This may offer a promising avenue for the development and clinical application of drugs targeting ischemic stroke.

## 2. Materials and Methods

### 2.1. Reagents

SHPL-49 was produced by Shanghai Hutchison Pharmaceuticals, lot number 3001430101. Edaravone was purchased from J&K Scientific Co., Ltd. (Beijing, China).

### 2.2. Animals

Male SD rats weighing 260–280 g were procured from Beijing Weitong Lihua Laboratory Animal Technology Co., Ltd. (Beijing, China). Male rats were chosen for the experiment because the androgen environment in males is relatively stable and does not fluctuate with the physiological cycle, which makes the experimental conditions easy to control. All the experimental protocols and procedures were approved by the Institutional Animal Care and Use Committee of Shanghai University of Traditional Chinese Medicine with the ethics number PZSHUTCM2303220004. All the experiments were conducted in accordance with The Guide for The Care and Use of Laboratory Animals published by the National Institutes of Health Animals, and ARRIVE guidelines were followed. The rats were housed in a temperature-controlled chamber maintained at 22–26 °C with relative humidity between 40% and 70% under a 12 h light/dark cycle. The rats were provided with unrestricted access to water and food.

### 2.3. Establishment of Rat Model of Permanent Middle Cerebral Artery Occlusion (pMCAO)

The rats were positioned on a heating pad and subjected to anesthesia using an induction dose of 3% and a maintenance dose of 2.5% isoflurane, ensuring body temperature was maintained at 37 °C ± 0.5 °C [30]. The left common carotid artery (CCA), internal carotid artery (ICA), and external carotid artery (ECA) were surgically exposed through a cervical incision. The proximal and distal CCA were ligated, while the distal ECA was also ligated. A silica-coated wire (Guangzhou Jialing Biotechnology Co., Ltd., Guangzhou, China) was carefully inserted through a small incision in the CCA and gradually advanced into the anterior end of the middle cerebral artery. The wound was sutured and disinfected after occlusion. During ligation, alterations in cerebral blood flow were monitored using a laser Doppler flow meter (Moor VSM, Axminster, UK). Success was defined as a reduction of 70% in ipsilateral cerebral blood flow compared to the contralateral hemisphere [27]. The survival rate of pMCAO rats was 80%. The physical and mental states of rats were closely monitored on a daily basis throughout the experiment. SHPL-49 and ED were administered via intravenous injection through the tail vein. After the completion of the drug intervention, the experimental rats were euthanized by bleeding from the abdominal aorta under deep anesthesia.

### 2.4. Cell Culture

The BV2 cells of microglia from mice were cultured in complete DMEM (HAKATA, China, A19008) supplemented with 10% fetal bovine serum (FBS) obtained from Hyclone, UT, USA, SH30071, and 1% penicillin–streptomycin. The highly differentiated PC-12 cells were acquired from Shanghai Zhong Qiao Xin Zhou Biotech Co., Ltd., Beijing, China. and cultured in complete DMEM. Each type of cell was cultured under a humidified atmosphere containing 5% CO_2_ at a temperature of 37 °C.

### 2.5. Establishment of Cell Model for Simulating Oxygen–Glucose Deprivation

The oxygen–glucose deprivation (OGD) technique was employed to simulate cerebral ischemia in cellular models. After washing the cells with phosphate-buffered saline (PBS) (HAKATA, Shanghai, China, A19712), the culture medium was substituted with sugar-free and serum-free 1640 medium (HAKATA, Shanghai, China, A19011) in the Model group. The drug administration groups were provided with a sugar-free, serum-free medium containing the drug instead of the media. The hypoxic tank was connected to a hypoxic apparatus to achieve hypoxia, and the gas composition was altered to 95% N_2_, 4% CO_2_, and 1% O_2_. Subsequently, the hypoxic tank was placed inside the cell culture chamber (Thermo, 4111FO, Waltham, MA, USA).

### 2.6. Establishment of Co-Culture Model Integrating BV2 Cells and PC-12 Cells

A co-culture cell model of BV2 and PC12 cells was established, with BV2 cells placed on the top layer of Transwell chambers (LABSELCT, Hefei, Anhui, China, 32023185X). Simultaneously, PC-12 cells within the chambers were maintained in DMEM and co-cultured for 2 days to ensure stability. Subsequently, the co-culture system was subjected to an OGD environment for further experiments.

### 2.7. Immunofluorescent Staining Assay

Immunofluorescence staining of brain sections: The brain paraffin sections were dewaxed and subjected to a gradient rehydration process using xylene and ethanol. Subsequently, they were blocked with 10% goat serum for 1 h at room temperature. The sections were subsequently incubated overnight at 4 °C with the designated primary antibodies as follows: P62/SQSTM1 (1:100, Boster Biological Technology, Wuhan, China, BM4385), LC3B (1:200, AiFang biologic, Changsha, Hunan, China, AF11004), and Iba1 (1:500, Servicebio, Wuhan, China, GB12105-100). After being washed with PBS, the sections were incubated for 2 h at room temperature with secondary antibodies against goat anti-mouse IgG H&L (Alexa Fluor^®^ 488) (1:500, Abcam, Cambridge, UK, ab150113) or donkey anti-rabbit IgG H&L (Alexa Fluor^®^ 555) (1:500, Abcam, Cambridge, UK, ab150074). After washing three times with PBS (Servicebio, Wuhan, China, G0002), the sections were treated with an anti-fluorescence quenching sealer (Beyotime, Shanghai, China, P0131) containing 4, 6-diamidino-2-phenylindole (DAPI) and covered with coverslips. Tissue sections were viewed using a laser confocal microscope (Nikon, Ti2, Otawara, Japan) [31]. Immunofluorescence staining of BV2 cells: The medium was discarded, and the cells were fixed with a 4% paraformaldehyde solution. Subsequently, the cells were permeated with 0.5% Triton X-100 (Adamas-beta^®^, Shanghai, China) and subsequently incubated with 5% bovine serum albumin (BSA, Beyotime, Shanghai, China) at room temperature for 30 min. After washing, the cells were incubated overnight at 4 °C with the subsequent primary antibodies, each of which possesses specific characteristics as described below: LC3 (1:1000, AiFang biologic, Hunan, China, AF11004) and NF-κB p65 (1:400, Cell Signaling Technology, Danvers, MA, USA, D14E12). After washing, the cells were incubated for 1 h at room temperature with goat anti-mouse IgG H&L (Alexa Fluor^®^ 488) (1:500, Abcam, Cambridge, UK, ab150113) or donkey anti-rabbit IgG H&L (Alexa Fluor^®^ 555) (1:500, Abcam, Cambridge, UK, ab150074) secondary antibodies. The nuclei were stained with DAPI and subsequently observed under a laser confocal microscope (Nikon, Otawara, Japan, Ti2).

### 2.8. Immunohistochemistry

Rat brain tissue sections were subjected to dewaxing and dehydration, followed by antigen retrieval using an antigen retrieval solution. After rinsing three times with PBS (Servicebio, Wuhan, China, G0002), the sections were incubated with 5% BSA (Beyotime, Shanghai, China, ST023) for one hour at room temperature. Subsequently, the sections were incubated overnight at 4 °C with primary antibodies against NF-κB (1:400, Cell Signaling Technology, MA, USA, D14E12) and IL-6 (Servicebio, Wuhan, China, GB11117-100). The following day, the sections were incubated with horseradish peroxidase (HRP)-conjugated goat anti-rabbit IgG (H + L) secondary antibody at room temperature for one hour. Diaminobenzidine was used for color development, and cell nuclei were counterstained with Harris hematoxylin. The sections were examined under an inverted fluorescence microscope (Nikon) using white light illumination. A quantitative analysis of the images was performed using ImageJ 1.8.0 software. For statistical analysis, at least three sections were randomly selected from each group, and 3–5 different regions were randomly chosen from each section.

### 2.9. Real-Time Quantitative PCR (RT-qPCR)

The total mRNA from BV2 cells was extracted using Trizol (Vazyme, Nanjing, Jiangsu, China, R401-01), and the concentrations of mRNA were determined using an ultramicro spectrophotometer (DeNovix, Wilmington, DE, USA, TFB-TIWI-01). After the removal of DNA and reverse transcription to generate the cDNA (Vazyme, Jiangsu, China, R223-01), real-time quantitative PCR (RT-qPCR) was performed using Vazyme’s kit (Vazyme, Jiangsu, China, R711-03). The data were analyzed by employing the 2^−ΔΔct^ method [32]. The PCR primers utilized in the experiment were as follows. β-actin: F: GGC-TGTATTCCCCTCCATCG, R: CCAGTTGGT-AACAATGCCATGT; IL-6: F: CTCTGCAAGAGACTTCCATCCAGT, R: CATTTCCACGATTTCCCAGAGA; IL-1β: F: CCTGTCCTGCGTGTTGAAAGA, R: GGGAACTGGGCAGACTCAAA;

iNOS: F: GGTATGCTGTGTT-TGGCCTT, R: GCAGCCTCTTGTCTTTGACC.

### 2.10. ELISA: Enzyme-Linked Immunosorbent Assay

The protein levels of the inflammatory factors IL-6 (Boster Biological Technology, Wuhan, China, EK0411), IL-1β (Boster Biological Technology, Wuhan, China, EK0394), and iNOS (JONLNBIO, Shanghai, China, JL20675) in the supernatants of treated BV2 cells were quantified using ELISA kits as per the manufacturer’s instructions.

### 2.11. Hoechst 33342 Staining Assay

The working solution of Hoechst 33342 (Servicebio, Wuhan, China, G1127) staining was added to the cell cultures at a volume of approximately one-tenth of the medium, ensuring the full coverage of the sample to be stained. The cultures were then incubated for 15 min in a 37 °C incubator. After the removal of the Hoechst 33342 staining working solution, the cell plates were washed 3 times with PBS and observed under a laser confocal microscope (Nikon, Otawara, Japan, Ti2).

### 2.12. Western Blot Assay

To extract the cytoplasmic and nuclear proteins, cells were lysed using a nuclear and cytoplasmic protein extraction kit (Beyotime, Shanghai, China, P0027) following the manufacturer’s instructions. Subsequently, the lysates were subjected to ultracentrifugation at 12,000× *g* for 10 min at 4 °C, and the resulting supernatants were collected as the cytoplasmic fraction. The pelleted nuclei were resuspended in a buffer containing 1 mM phenyl methane sulfonyl fluoride (PMSF, Beyotime, Shanghai, China, ST505). After incubation at 4 °C for 30 min, the lysates were centrifuged, and the supernatants, which contained the nuclear proteins, were stored at −80 °C. Whole-cell extracts were obtained as previously described. Total protein samples from BV2 and PC12 cells, as well as rat brain tissues, were extracted using RIPA lysis buffer (Beyotime, Shanghai, China, P0013B), and protein concentrations were measured using a BCA kit (Beyotime, Shanghai, China, P0010).

The protein samples were separated using SDS-PAGE and transferred to polyvinylidene difluoride membranes (Bio-Rad, Hercules, CA, USA, 1620177). Membranes were incubated with 5% nonfat milk (Beyotime, Shanghai, China, P0216) or BSA (Beyotime, Shanghai, China, ST023) followed by overnight incubation at 4 °C with the following primary antibodies: NF-κB (1:1000, Cell Signaling Technology, MA, USA, D14E12), p-IKKβ (1:1000, UpingBio, Hangzhou, Zhejiang, China, YP-Ab-14443), IKKβ (1:1000, Boster, Wuhan, China, BM4875), p-IκBα (1:1000, UpingBio, Zhejiang, China, YP-Ab-01253), IKBα (1:1000, UpingBio, Zhejiang, China, YP-Ab-01830), P62/SQSTM1 (1:1000, Boster, Wuhan, China, BM4385), LC3B (1:200, PTMab, Zhejiang, China, PTM-6384), ATG5 (1:1000, Cell Signaling Technology, MA, USA, D5F5U), Bclin1 (1:1000, Cell Signaling Technology, MA, USA, D40C5), LAMP2 (1:200, UpingBio, Zhejiang, China, YP-Ab-14094), Bax (1:1000, Cell Signaling Technology, MA, USA, 14796), Bcl-2 (1:1000, AiFang biologic, Hunan, China, AF0060), Cleaved caspase-3 (1:1000, Cell Signaling Technology, MA, USA, 9661S), and Cleaved caspase-9 (1:1000, Cell Signaling Technology, MA, USA, 9507S). The membranes were incubated with HRP-conjugated goat anti-rabbit IgG (H + L) (1:5000, Beyotime, Shanghai, China, A0208) or goat anti-mouse IgG (H + L) (1:5000, Beyotime, Shanghai, China, A0216) secondary antibodies for 2 h at room temperature. Immunolabeling was detected using ECL chemiluminescence (Meilunbio, Dalian, China, MA0186). Immunoblots were semi-quantitatively analyzed using ImageJ software (1.52a) [33].

### 2.13. Cell Viability Assay

The MTT reduction assay assessed the viability of PC12 cells. Briefly, the cells were rinsed with phosphate-buffered saline (PBS, Servicebio, Wuhan, China, G0002) and then incubated with a 5 mg/mL MTT reagent for 3 h at 37 °C. After removing the medium, cell lysis was performed using 1 mL of dimethyl sulfoxide, and the absorbance was read at 540 nm using an enzyme-labeled instrument (Thermo Fisher Scientific, Waltham, MA, USA, Multiskan GO).

### 2.14. Analysis by Transmission Electron Microscope

The brain tissues were sectioned into 1 mm^3^ pieces and fixed in a solution of 2.5% glutaraldehyde for 24 h, followed by post-fixation with 1% osmium tetroxide for 3 h. Subsequently, the samples were dehydrated using an ethanol gradient (50%, 70%, 90%, and finally 100%) for a duration of 10 min each. After embedding the samples in resin, they were sliced to a thickness of approximately 50–60 nm. The resulting sections were stained with uranyl acetate and lead citrate before being examined under a transmission electron microscope (JEOL, Tokyo, Japan, JEM-F200).

### 2.15. Adenovirus Infection and the Quantification of mRFP-GFP-LC3 Dots

Initially, BV2 cells were cultured in 12-well plates and subsequently infected with mRFP-GFP-LC3 adenovirus (HANBIO, Shanghai, China). After infection, the cells were treated with various drugs. Nuclear staining was performed using DAPI, and visualization was achieved through confocal microscopy (Nikon, Otawara, Japan, Ti2). The localization of LC3 was monitored using the mRFP tag, and a decrease in GFP expression indicated the fusion of autophagosomes with lysosomes and the eventual formation of autolysosomes. A change in pH results in the quenching of GFP fluorescence, leaving only detectable red fluorescence. During microscopic imaging, the red and green fluorescence images are merged, with yellow dots indicating autophagosomes and red dots indicating autophagolysosomal. To quantify the intensity of autophagic flux, the number of yellow and red dots was counted.

### 2.16. Lyso-Tracker Green Staining Assay

The presence of lysosomes was assessed by performing Lyso-Tracker Green staining (Servicebio, Wuhan, China, G1722) following the supplier’s instructions. The cells were incubated with Lyso-Tracker Green (50 nM) at 37 °C for 30 min. Subsequently, the images were captured using confocal microscopy (Nikon, Otawara, Japan, Ti2) [34].

### 2.17. Statistical Analysis

A statistical analysis of the data was performed using GraphPad Prism 9.0 software, with the results presented as the mean ± standard deviation. A one-way or two-way analysis of variance (ANOVA) was utilized for statistical testing, and a significance level of *p* < 0.05 was applied.

### 2.18. Statement of Ethics

The protocols and procedures involving animals in the experiment were all approved by the Ethics Committee of Shanghai University of Traditional Chinese Medicine after ethical review.

## 3. Results

### 3.1. SHPL-49 Exerts Anti-Inflammatory Effects by Suppressing the NF-κB Signaling Pathway

**Figure 1:**
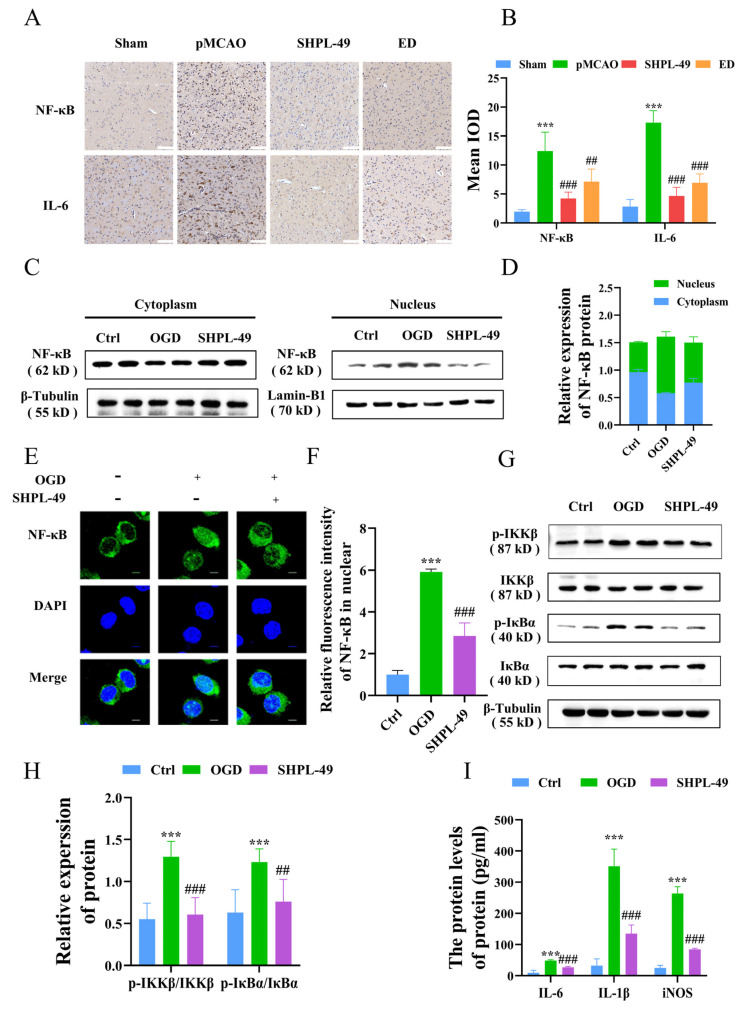
SHPL-49 exerts anti-inflammatory effects by suppressing the NF-κB signaling pathway. (**A**) Immunohistochemical staining images of NF-κB and IL-6 in brain tissue sections from rats subjected to pMCAO and treated with SHPL-49 (15 mg/kg) and ED (7.5 mg/kg) for three days, Scale = 100 μm. (**B**) The IODs for NF-κB and IL-6 immunohistochemistry in brain tissue sections for each treatment group. (**C**) Representative Western blot of NF-κB in the nucleus and cytoplasm of OGD-BV2 cells after treatment with SHPL-49 (200 μM). (**D**) Quantification of the Western blot results. (**E**) Immunofluorescence staining pattern of NF-κB in OGD-BV2 cells following treatment with SHPL-49 (200 μM); Scale = 10 μm. (**F**) Quantification of the average fluorescence intensity of NF-κB in the nucleus of BV2 cells. (**G**) Representative Western blot of p-IKKβ, IKKβ, p-IκBα, and IκBα proteins in OGD-BV2 cells after treatment with SHPL-49 (200 μM). (**H**) Quantification of the Western blot results. (**I**) The protein expression levels of inflammatory factors IL-6, IL-1β, and iNOS in the supernatant of OGD-BV2 cells following treatment with SHPL-49 (200 μM). Data are presented as means ± SD with *n* = 6 per group. *** *p* < 0.001 vs. Ctrl; ## *p* < 0.01, ### *p* < 0.001 vs. OGD.

To investigate the association between the inhibitory effect of SHPL-49 on neuroinflammation and the NF-κB signaling pathway, we established a pMCAO model and administered continuous intravenous injections of SHPL-49 (15 mg/kg) for 3 days, starting at 30 min post-operation. Edaravone (ED, 7.5 mg/kg), a well-established free radical scavenger with recognized anti-inflammatory properties, was used as the positive control. The effect of SHPL-49 on the expression of NF-κB and inflammatory factor IL-6 in the brain tissue of pMCAO rats was assessed through immunohistochemical staining (Figure 1A,B). The mean integral optical density (IOD) for NF-κB and IL-6 increased significantly in the Model group compared to the Sham Group. Following 3 days of SHPL-49 (15 mg/kg) or ED (7.5 mg/kg) treatment, the mean IODs for NF-κB and IL-6 were significantly lower when compared with those in the Model group. To further investigate the inhibitory effect of SHPL-49 on neuroinflammation, we established an in vitro OGD model using BV2 cells. The impact of SHPL-49 on NF-κB signaling pathway in OGD-BV2 cells was evaluated through Western blot and immunofluorescence staining (Figure 1C–E). The nuclear proportion of NF-κB protein levels exhibited a significant increase in the OGD-BV2 cell model compared to the Control group. Treatment with SHPL-49 significantly attenuated the alterations in NF-κB levels induced by OGD. To further validate this, we utilized Western blotting to investigate the effects of SHPL-49 on the NF-κB signaling pathway (Figure 1F,G). The phosphorylation levels of IKKβ and IκBα were found to be elevated in the OGD-BV2 group compared to the Control group, whereas SHPL-49 exhibited a significant reduction in the phosphorylation levels of IKKβ and IκBα when compared to the Model group. These findings suggest that SHPL-49 effectively inhibits the activation of the NF-κB signaling pathway. Subsequently, we conducted further investigations to examine the regulatory effects of SHPL-49 on the expression levels of IL-6, IL-1β, and iNOS proteins in BV2 cells (Figure 1H). The levels of IL-6, IL-1β, and iNOS proteins in the supernatant were significantly elevated in the OGD-BV2 group compared to the Control group, whereas treatment with SHPL-49 effectively attenuated the expression levels of IL-6, IL-1β, and iNOS in the supernatant.

### 3.2. SHPL-49 Attenuates Excessive Autophagy in the Brain Tissue of Rats with pMCAO

**Figure 2:**
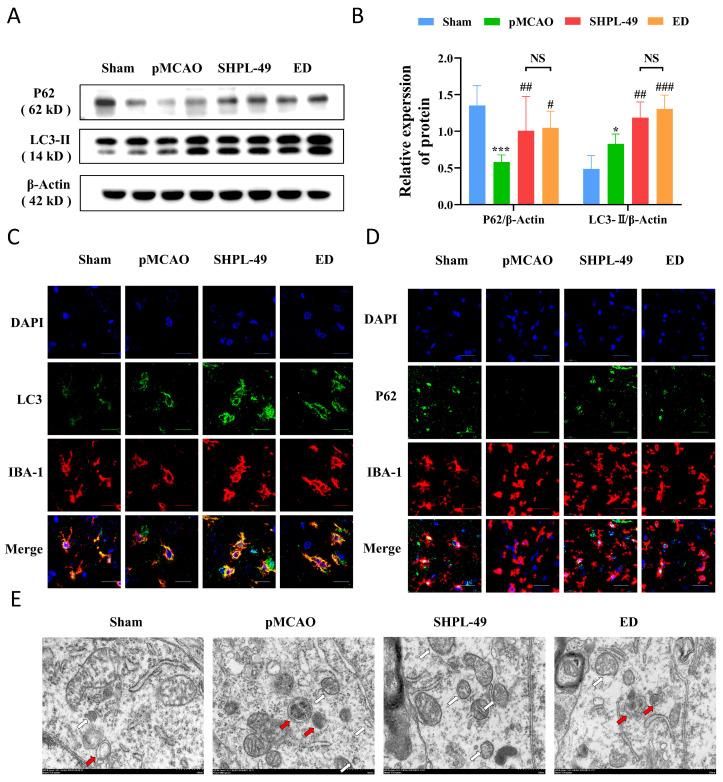
SHPL-49 attenuates excessive autophagy in the brain tissue of rats with pMCAO. (**A**) The Western blot analysis of P62 and LC3-II protein expression in rats with pMCAO treated with SHPL-49 (15 mg/kg) and ED (7.5 mg/kg) for three days. (**B**) Quantification of the Western blot results. (**C**,**D**) Immunofluorescence staining images of IBA-1-P62 and IBA-1-LC3II in brain tissue sections of rats subjected to pMCAO treated with SHPL-49 (15 mg/kg) and ED (7.5 mg/kg) for three days. Scale = 50 μm (**E**) Revealing autophagosomes in brain tissue through transmission electron microscopy observation. Data are presented as means ± SD, with *n* = 3 per group. Scale = 500 nm. * *p* < 0.05, *** *p* < 0.001 vs. Sham; # *p* < 0.05, ## *p* < 0.01, ### *p* < 0.001 vs. pMCAO.; NS, Not Significant.

To investigate the impact of SHPL-49 on autophagy in the pMCAO rats, we employed the established pMCAO model and administered intravenous injections of SHPL-49 (15 mg/kg) continuously for 3 days starting at 30 min post-operation. Western blot analysis was utilized to assess the expression levels of key autophagy proteins, P62 and LC3-II, within brain tissue (Figure 2A,B). After stroke induction, the Model group exhibited a significant decrease in P62 expression compared to the Sham group, while there was a significant increase in LC3-II expression. Notably, treatment with either 15 mg/kg of SHPL-49 or 7.5 mg/kg of ED resulted in a prominent increase in the levels of both P62 and LC3-II. To further validate whether SHPL-49 modulates neuroinflammation via the regulation of microglial-mediated autophagy, we conducted double-label immunofluorescence experiments (Figure 2C,D). The expression of P62 in microglia was significantly decreased in the Model group compared to the Sham group, whereas LC3 expression was significantly elevated. Treatment with either 15 mg/kg of SHPL-49 or 7.5 mg/kg of ED markedly increased both P62 and LC3 levels in microglia. Additionally, for a more detailed investigation into autophagic flux, transmission electron microscopy was employed (Figure 2E). The autophagosome, observed under the transmission electron microscope, presents as a spherical structure with a double membrane and contains organelles and proteins specifically targeted for degradation. It is clearly indicated by a white arrow in the accompanying figure. Conversely, the autolysosome is formed through the fusion of an autophagosome and a lysosome, exhibiting a monolayer membrane structure that encapsulates degraded cytoplasmic components. This feature is marked by a red arrow in the figure. The Model group exhibited an elevated quantity of autophagosomes and autolysosomes in comparison to the Sham group, whereas in the SHPL-49 treatment group, there was an increase in the number of autophagosomes but a decrease in autolysosomes. These results suggest that SHPL-49 may impede autophagic flux, resulting in an upregulation of P62 and LC3 protein expression while concurrently suppressing excessive autophagy activation in microglia.

### 3.3. SHPL-49 Inhibits Excessive Autophagy of OGD-BV2 Cells

**Figure 3:**
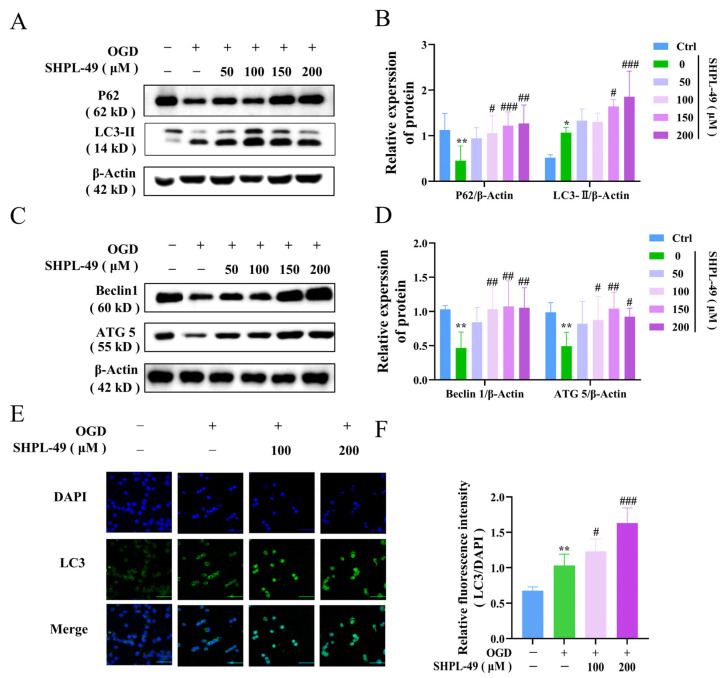
SHPL-49 inhibits excessive autophagy of OGD-BV2 cells. (**A**) Representative Western blot of P62 and LC3-II in OGD-BV2 cells treated with varying concentrations of SHPL-49. (**B**) Quantification of the Western blot results. (**C**) Representative Western blot of Beclin-1 and ATG5 in OGD-BV2 cells treated with different concentrations of SHPL-49. (**D**) Quantification of the Western blot results. (**E**) Immunofluorescence staining of LC3 in OGD-BV2 cells treated with SHPL-49 (100 μM, 200 μM). (**F**) Quantification of the average fluorescence intensity of LC3. Data are presented as means ± SD, with *n* = 6 per group. * *p* < 0.05, ** *p* < 0.01 vs. Ctrl; # *p* < 0.05, ## *p* < 0.01, ### *p* < 0.001 vs. OGD. Scale = 100 μm.

To validate the regulatory role of SHPL-49 in microglial autophagy, we established an in vitro model of oxygen–glucose deprivation (OGD) using BV2 cells. The impact of varying concentrations of SHPL-49 on the expression of autophagy proteins in OGD-BV2 cells was evaluated through Western blot analysis (Figure 3A–D). Compared to the Control group, the OGD-BV2 group exhibited a significant decrease in the expression levels of P62, ATG5, and Beclin-1 proteins. Importantly, treatment with 100 µM, 150 µM, and 200 µM of SHPL-49 effectively reversed the protein expression alterations induced by OGD in BV2 cells. Furthermore, the OGD-BV2 group exhibited a significant elevation in LC3-II protein. Moreover, treatment with 150 µM and 200 µM of SHPL-49 further increased the expression of the LC3-II protein. The observed elevation can be attributed to an excessive induction of autophagy in BV2 cells during OGD, leading to the accumulation of LC3-II protein. However, treatment with SHPL-49 appeared to inhibit autophagic flux, resulting in a further augmentation of LC3-II levels. To further substantiate these findings, immunofluorescence experiments were conducted (Figure 3E,F). In the OGD-BV2 group, a granular accumulation of the LC3 protein was observed. Conversely, in the SHPL-49 treatment group, a significant enhancement in green fluorescence intensity representing granular LC3 protein was significantly noted, suggesting that SHPL-49 may promote the aggregation of LC3 puncta by further impeding autophagy.

### 3.4. SHPL-49 Impedes the Autophagic Flow in OGD-BV2 Cells Through the Inhibition of Lysosomal Activity

**Figure 4:**
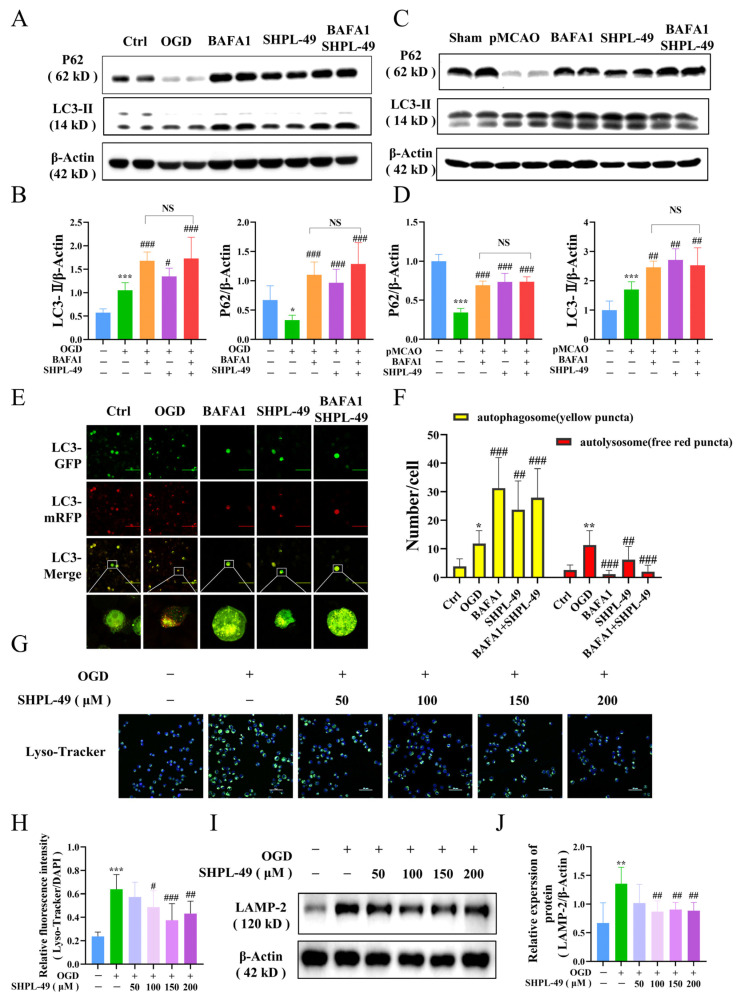
SHPL-49 impedes the autophagic flow in OGD-BV2 cells through the inhibition of lysosomal activity. (**A**) Representative Western blot of P62 and LC3-II in OGD-BV2 cells treated with SHPL-49 (200 μM) and BAFA1 (200 nM), Scale = 100 μm. (**B**) Quantification of the Western blot results. (**C**) Representative Western blot of P62 and LC3-II in brain tissue from pMCAO rats treated with SHPL-49 (15 mg/kg) and BAFA1 (1 mg/kg) three days post-stroke. (**D**) Quantification of the Western blot results. (**E**) Representative fluorescence images of OGD-BV2 infected with mRFP-GFP-LC3 adenovirus. (**F**) Quantification of red and yellow fluorescence spots in OGD-BV2 cells. (**G**) Representative immunofluorescence staining of lysosomes in OGD-BV2 cells treated with SHPL-49 (100 μM, 200 μM); Scale = 50 μm. (**H**) The quantification of average fluorescence intensity for Lyso-Tracker. (**I**) Representative Western blot of LAMP-2 in OGD-BV2 cells treated with different concentrations of SHPL-49. (**J**) Quantification of the Western blot results. Data are presented as means ± SD, *n* = 6 per group. * *p* < 0.05, ** *p* < 0.01, *** *p* < 0.001 vs. Ctrl; # *p* < 0.05, ## *p* < 0.01, ### *p* < 0.001 vs. OGD; NS, Not Significant.

To further investigate the effects of SHPL-49 on autophagy, we utilized the autophagy inhibitor BAFA1 to monitor autophagic flux. Western blotting was employed to assess the expression of key autophagy proteins, P62 and LC3-II, in BV2 cells and brain tissue (Figure 4A–D). Compared to the Control group, there was a significant decrease in P62 expression and a notable increase in LC3-II expression observed in both the OGD-BV2 cell and pMCAO rat Model groups. Compared with the Model group, treatment with autophagy inhibitors BAFA1 and SHPL-49 significantly upregulated the protein levels of P62 and LC3-II. Notably, the addition of SHPL-49 in conjunction with BAFA1 did not result in significant changes in the expression levels of LC3-II and P62 when compared to the BAFA1 alone group, suggesting that SHPL-49 does not induce autophagic vesicle formation. Subsequently, BV2 cells were transfected with a dual-labeled LC3 adenovirus, employing RFP and GFP as simultaneous markers for LC3 proteins. The RFP emits red fluorescence, while the GFP emits green fluorescence. Upon autophagosome formation, these two fluorophores co-localize on the autophagosome, resulting in the appearance of yellow, fluorescent spots. Due to the rapid quenching of GFP fluorescence in acidic environments, when autophagosomes fuse with lysosomes to form autolysosomes and lower the pH to 4, GFP is quenched, and only RFP stably emits red fluorescence. Therefore, the yellow spots represent autophagosomes, and the red spots indicate autolysosomes (Figure 4E,F). The OGD-BV2 group exhibited a significant increase in both autophagic bodies and autolysosomes compared to the Control group. Conversely, the SHPL-49 treatment group demonstrated a decrease in autolysosome counts along with a significant increase in the number of autophagic bodies, resembling the pattern observed with the autophagy inhibitor BAFA1. Collectively, these findings suggest that SHPL-49 may exert inhibitory effects on autophagy by impeding the fusion process between autophagic bodies and autolysosomes. Subsequently, to assess the impact of SHPL-49 on lysosomal function, we utilized Lyso-Tracker staining and Western blot analysis to examine the expression of LAMP-2, a key protein associated with lysosomes, in BV2 cells (Figure 4G–J). The lysosomal protein levels and fluorescence in OGD-BV2 cells exhibited an enhancement; however, treatment with SHPL-49 led to a significant reduction in both lysosomal fluorescence intensity and protein expression. These results suggest that SHPL-49 may inhibit the fusion of autophagic bodies with lysosomes by suppressing lysosomal activity, thereby hindering the flow of autophagy.

### 3.5. SHPL-49 Reduces Inflammation by Suppressing Excessive Autophagy and NF-κB Signaling in OGD-BV2 Cells

**Figure 5:**
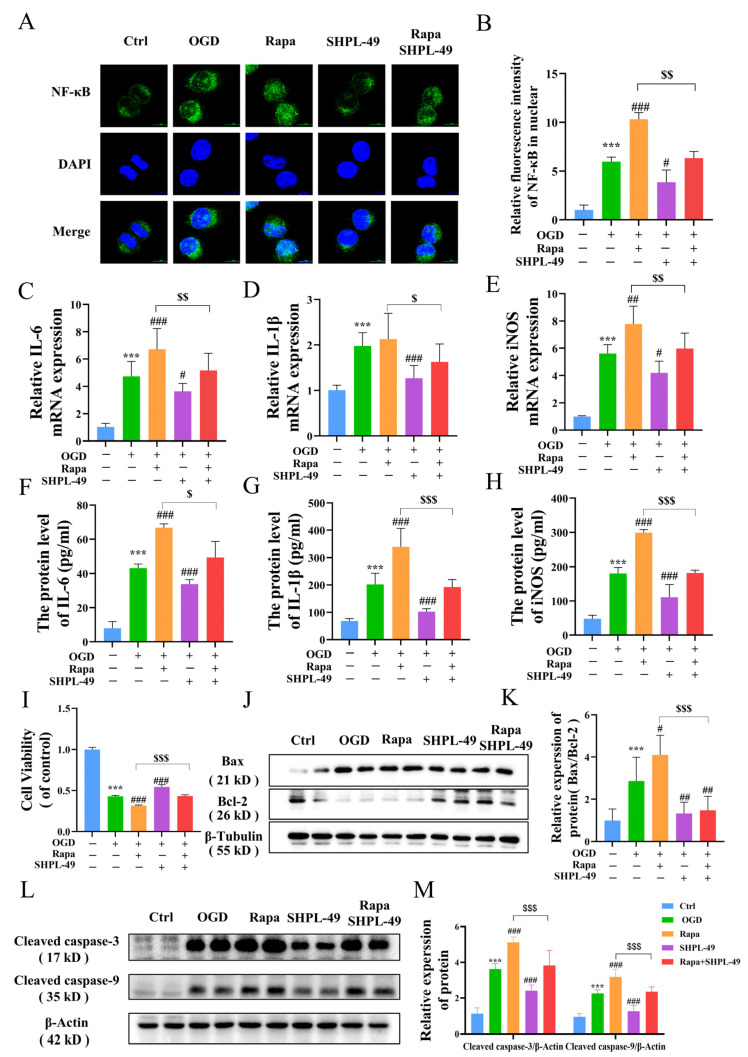
SHPL-49 reduces inflammation by suppressing excessive autophagy and NF-κB signaling in OGD-BV2 cells. (**A**) Immunofluorescence staining of NF-κB in OGD-BV2 cells treated with SHPL-49 (200 μM) and Rapa (100 nM); Scale = 10 μm. (**B**) Quantification of the average fluorescence intensity of NF-κB in the nucleus of OGD-BV2 cells. (**C**–**E**) The mRNA levels of IL-6, IL-1β, and iNOS in OGD-BV2 cells treated with SHPL-49 (200 μM) and Rapa (100 nM). (**F**–**H**) The protein levels of IL-6, IL-1β, and iNOS in the supernatants of OGD-BV2 cells treated with SHPL-49 (200 μM) and Rapa (100 nM). (**I**) The viability of PC12 cells was assessed by co-culturing them with OGD-BV2 cells treated with SHPL-49 (200 μM) and Rapa (100 nM) with PC12 cells. (**J**) Representative Western blot of BAX and Bcl-2 in PC12 cells after co-culture treatment. (**K**) Quantification of the Western blot results. (**L**) Representative Western blot of Cleaved caspase-3 and Cleaved caspase-9 in PC12 cells after co-culture treatment. (**M**) Quantification of the Western blot results. Data are presented as means ± SD, *n* = 6 per group. *** *p* < 0.001 vs. Ctrl; # *p* < 0.05, ## *p* < 0.01, ### *p* < 0.001 vs. OGD; $ *p* < 0.05, $$ *p* < 0.01, $$$ *p* < 0.001 vs. Rapa. Scale = 10 μm.

To determine whether the neuroprotective effects of SHPL-49 on OGD-BV2 cells are mediated through the modulation of autophagy, we conducted experiments utilizing the autophagy inducer Rapa. Compared to the OGD-BV2 group, treatment with Rapa significantly upregulated NF-κB protein expression within cellular nuclei. Moreover, the co-treatment of Rapa and SHPL-49 resulted in a notable reduction in nuclear NF-κB expression compared to the Rapa-only group, indicating that SHPL-49 may exert inhibitory effects on autophagy and NF-κB activation (Figure 5A,B). The mRNA and protein levels of inflammatory factors, including IL-6, IL-1β, and iNOS in BV2 cells, were further examined (Figure 5C–H). Our findings demonstrate that the administration of SHPL-49 resulted in a significant reduction in the expression levels of these inflammatory factors compared to the OGD-BV2 group. Conversely, treatment with Rapa significantly enhanced both the mRNA and protein levels of inflammatory factors, indicating that autophagy induction can result in the upregulated expression of these factors. The co-treatment of Rapa and SHPL-49 resulted in a decreased expression of inflammatory factors compared to the group treated with Rapa alone while exhibiting increased expression levels compared to the group treated with SHPL-49 alone. The collective findings suggest that SHPL-49 may exert its anti-inflammatory effects in OGD-BV2 cells by suppressing autophagy. Subsequently, we established an in vitro co-culture system of BV2 and PC12 neuronal cells to mimic the impact of microglia on PC12 cells (Figure 5I–K). The viability of PC12 cells following co-culture was assessed using the MTT assay. Western blot analysis was employed to quantify the protein ratios of pro-apoptotic Bax and anti-apoptotic Bcl-2, as well as the expression levels of apoptotic markers Cleaved caspase-3 and Cleaved caspase-9. Compared with the Control group, PC12 cells exposed to oxygen–glucose deprivation (OGD) exhibited a significant reduction in viability. Additionally, the Bax/Bcl-2 ratio was markedly elevated, and the expression levels of Cleaved caspase-3 and Cleaved caspase-9 were significantly increased. Treatment with the autophagy inducer rapamycin (Rapa) further exacerbated these effects compared to the OGD group, leading to a substantial decrease in cell viability, an increase in the Bax/Bcl-2 ratio, and higher expression of Cleaved caspase-3 and Cleaved caspase-9. Conversely, the administration of SHPL-49 significantly improved PC12 cell viability, reduced the Bax/Bcl-2 ratio, and decreased the expression of Cleaved caspase-3 and Cleaved caspase-9. Moreover, combined treatment with rapamycin and SHPL-49 synergistically enhanced cell viability, lowered the Bax/Bcl-2 ratio, and reduced the expression of Cleaved caspase-3 and Cleaved caspase-9 compared to rapamycin treatment alone. These findings suggest that SHPL-49 enhances the survival of PC12 cells during co-culture with OGD-treated BV2 cells by inhibiting excessive autophagy in BV2 cells and reducing the expression of apoptotic proteins in PC12 cells, thereby protecting PC12 cells. Furthermore, the experimental results indicate that SHPL-49 mitigates the inflammatory response by suppressing autophagy and the nuclear factor kappa B (NF-κB) signaling pathway in OGD-treated BV2 cells, thus providing protection for PC12 cells.

## 4. Discussion

The incidence and mortality rates of ischemic stroke are considerably high, rendering it a prevalent disease that exerts a significant impact on human health [35]. Inflammation subsequent to an ischemic stroke extends beyond the surrounding ischemic lesions, disseminating throughout the entire brain and persisting for an extended duration. This sustained inflammatory response profoundly influences the pathological and physiological alterations in cerebral tissue following a stroke [36].

Microglial cells, serving as the primary mediators of neuroimmune inflammation in the brain following a stroke, play pivotal roles in immune recruitment, regulation, inflammation modulation, phagocytosis facilitation, and vascular repair promotion. Therefore, they are indispensable for the development of stroke therapeutics [37]. In this study, we demonstrated that SHPL-49 effectively inhibits neuroinflammation by modulating microglial cell autophagy and exerts significant neuroprotective effects. Our findings indicate that SHPL-49 suppresses the autophagic process in microglial cells within the brain tissue of pMCAO rats, thereby attenuating the NF-κB signaling pathway and ameliorating neuroinflammatory responses. Furthermore, cell experiments further validated the inhibitory effects of SHPL-49 on lysosomal activity in OGD-BV2 cells, as well as its ability to impede the fusion between autophagosomes and lysosomes. Ultimately, this leads to the suppression of excessive autophagy in OGD-BV2 cells, resulting in the reduced activation of the NF-κB signaling pathway and decreased protein expression levels of inflammatory factors. Additionally, by inhibiting autophagy, SHPL-49 enhances the viability of co-cultured PC12 cells, thereby contributing to its neuroprotective effects against ischemic stroke. Nevertheless, our study possesses certain limitations. Given that BV2 cells are immortalized cell lines, they cannot fully replicate the physiological and functional intricacies of primary microglial cells. Previous studies utilizing RNA-seq have compared transcriptomic alterations in BV2 microglial cell lines and primary microglia (PM) following lipopolysaccharide (LPS) stimulation [38]. These studies revealed that PM exhibited a more robust response to LPS, with a significantly greater number of altered transcripts compared to the BV2 cell line. Consequently, future research endeavors will aim to further validate the efficacy of SHPL-49 on primary glial cells to comprehensively and accurately assess its potential as a therapeutic agent for ischemic stroke.

The NF-κB signaling pathway serves as the central mediator of inflammatory reactions, playing important roles in cellular responses to various stimuli [39]. Upon stimulation by hypoxia, inflammatory cytokines, microbial products, and other factors, signaling molecules, including the IKK complex, become activated. This activation leads to the phosphorylation and degradation of IκB proteins, resulting in the release and activation of NF-κB. The liberated NF-κB translocates from the cytoplasm to the nucleus, where it activates the transcription of target genes such as IL-1β and IL-6, thereby augmenting the levels of inflammatory factors [40]. In this study, we observed a significant increase in the protein levels of NF-κB in the rat pMCAO model. Additionally, the NF-κB signaling pathway was found to be activated in OGD-BV2 cells. Moreover, there was an elevation in the phosphorylation ratios of IKK and IκB-α proteins, along with a substantial rise in the protein levels of inflammatory factors IL-6, IL-1β, and iNOS. The administration of SHPL-49 significantly suppressed the activation of the NF-κB signaling pathway in rat pMCAO and OGD-BV2 cells, leading to a reduction in the expression levels of inflammatory factors. However, the precise mechanism underlying SHPL-49’s inhibition of neuroinflammation remains elusive and warrants further investigation.

Autophagy is a vital cellular process that facilitates the degradation or elimination of damaged proteins and organelles within the cytoplasm through the lysosomal degradation pathway [41]. Autophagy represents an intricate mechanism initiated by genetic regulation involving the gradual formation of double-membrane structures. The Beclin-1/VPS34 complex subsequently facilitates the continuous elongation of autophagic double-membrane structures, while the ATG12-ATG5 complex associates with ATG16 to accomplish aggregation [42]. The aggregated complexes fuse with autophagic vesicles to form autophagosomes. During the formation of autophagosomes, the Beclin-1 protein promotes the conversion of LC3-I, a key autophagy protein, into LC3-II [43]. Consequently, there exists a significant correlation between the abundance of LC3-II and the quantity of autophagic bodies. However, the quantification of LC3-II at a specific point is insufficient to determine the autophagic flux, which serves as a more comprehensive indicator of overall autophagic degradation rather than solely reflecting autophagosome formation [44]. The STX17 complex ultimately combines with SNAP29 and VAMP8 to form the SNARE complex, which subsequently translocates to the membrane of autophagic bodies. This facilitates the fusion between lysosomes and autophagic bodies, leading to the formation of autolysosomes [45].

The protein P62 functions as an autophagic adapter and plays a crucial role in the degradation of proteins. During the process of autophagic lysosomal degradation, P62 binds to substrates and subsequently undergoes degradation by proteases. Consequently, an elevation in the level of P62 protein is commonly regarded as an indicator of suppressed autophagic activity [45]. To investigate the potential of SHPL-49 in modulating neuroinflammation through autophagy regulation, we examined its impact on cellular autophagic proteins and further assessed its effect in the presence of the autophagy inhibitor BAFA1. Our study revealed that treatment of OGD-BV2 cells with SHPL-49 resulted in elevated levels of LC3-II, P62, ATG5, and Beclin-1 proteins, potentially attributed to the inhibition of autophagic body degradation and subsequent protein accumulation. Subsequently, we conducted a further analysis of autophagy by introducing the autophagy inhibitor BAFA1. The fusion of autophagic bodies with lysosomes was inhibited by BAFA1. However, the co-administration of BAFA1 and SHPL-49 did not significantly alter the expression of LC3-II and P62 proteins compared to the BAFA1 group, thereby further confirming the inhibitory effects of SHPL-49 on autophagy. The inhibitory effects of SHPL-49 on lysosome activity were subsequently confirmed through the detection of lysosomal-associated membrane protein 2 (LAMP-2) and the visualization of lysosomes using Lyso-Tracker fluorescent dye, thereby providing further evidence for the inhibition of autophagic body fusion with lysosomes by SHPL-49.

The mounting evidence suggests an intricate network connection between autophagy and NF-κB, with the influence between them remaining contentious and contingent upon cell type and stimuli. The study conducted by Mingxia Zhou et al. [46] used in vitro experiments to demonstrate that lipopolysaccharide (LPS) suppresses autophagy in colitis through the TLR4-MyD88-MAPK pathway, thereby orchestrating downstream NF-κB activation and resulting in the generation of proinflammatory cytokines and oxidative stress. Alfredo Criollo et al. [47] reported that the activation of the NF-κB signaling pathway necessitates autophagic stimulation. Therefore, in order to investigate the involvement of autophagy in SHPL-49-mediated anti-inflammatory effects, an autophagy inducer, Rapa, was incorporated into the experiments. The findings demonstrated that the autophagy inducer Rapa significantly augmented NF-κB activation within the nucleus and enhanced the expression of inflammatory cytokines IL-6, IL-1β, and iNOS. However, the co-administration of SHPL-49 and Rapa effectively inhibits NF-κB activation and attenuates inflammatory response induced by Rapa. In conclusion, the inhibition of autophagy can reduce the inflammatory response triggered by ischemic stroke, while our compound SHPL-49 demonstrates its protective effects against ischemic stroke through microglial cell autophagy inhibition and the alleviation of neuroinflammation.

**Figure 6:**
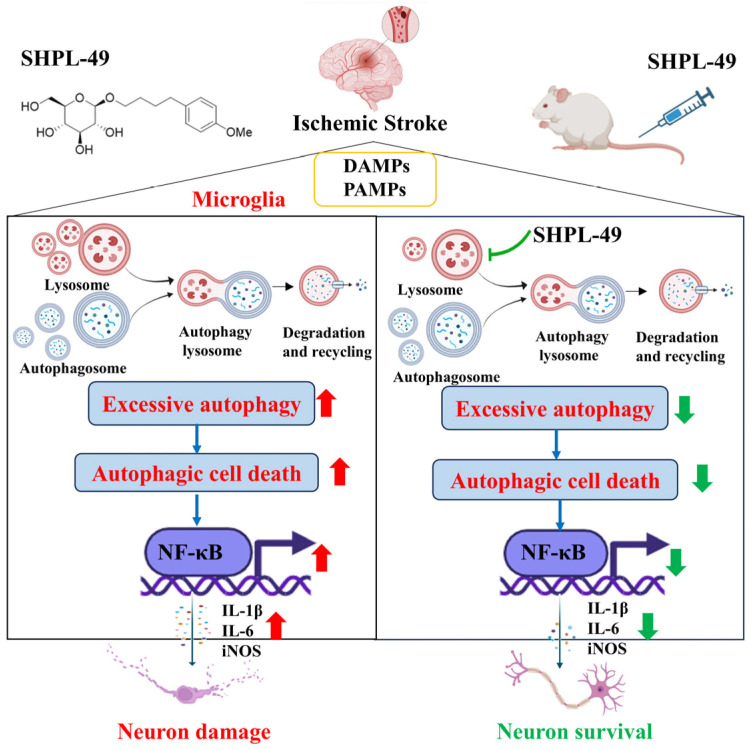
Schematic representation of mechanism by which SHPL-49 impedes microglial autophagy and mitigates neuroinflammation through inhibition of lysosomal activity.

Based on previous pharmacological studies investigating the neuroprotective effects of SHPL-49 in rats with cerebral ischemia, we further explored the effects of SHPL-49 on microglial autophagy and neuroinflammation. Overall, as shown in the Schematic diagram (Figure 6), the findings suggest that SHPL-49 hinders autophagy by suppressing lysosomal activity and the fusion between autophagosomes and lysosomes, thereby suppressing autophagy and reducing NF-κB pathway activation and exerting an anti-neuroinflammatory effect. This study provides valuable experimental data for researching treatments targeting neuroinflammation in ischemic stroke while also offering new potential active compounds for treating this condition and presenting a novel approach to developing and applying neuroprotective agents.

## Data Availability

The raw data supporting the conclusions of this article will be made available by the authors upon request.

## Abbreviations

The following abbreviations are used in this manuscript:

ATG-5	Autophagy-related protein 5
CCA	Common carotid artery
ECA	External carotid artery
ED	Edaravone
ELISA	Enzyme-linked immunosorbent assay
IBA-1	Ionized calcium-binding adaptor molecule 1
ICA	Internal carotid artery
IL-1β	Interleukin-1 beta
IL-6	Interleukin 6
IKK	IκB kinase
LAMP2	lysosome-Associated Membrane Protein 2
LC3	Including Light Chain 3
NF-κB	Nuclear factor kappa-B
OGD	Oxygen–glucose deprivation
pMCAO	permanent Middle Cerebral Artery Occlusion
P62	p62/sequestosome 1
PM	Primary microglia
RAPA	Rapamycin
TEM	Transmission Electron Microscopy
